# Inhaled Corticosteroids

**DOI:** 10.3390/ph3030514

**Published:** 2010-03-08

**Authors:** Peter J. Barnes

**Affiliations:** National Heart and Lung Institute, Imperial College, London, UK; Email: p.j.barnes@imperial.ac.uk; Tel.: +44 207-351-8174; Fax: +44 207-351-5675.

**Keywords:** glucocorticoid receptor, nuclear factor-κB, inflammatory gene, histone deacetylase, eosinophil, epithelial cell, long-acting β_2_-agonist, inflammation, pneumonia

## Abstract

Inhaled corticosteroids (ICS) are the most effective controllers of asthma. They suppress inflammation mainly by switching off multiple activated inflammatory genes through reversing histone acetylation *via* the recruitment of histone deacetylase 2 (HDAC2). Through suppression of airway inflammation ICS reduce airway hyperresponsiveness and control asthma symptoms. ICS are now first-line therapy for all patients with persistent asthma, controlling asthma symptoms and preventing exacerbations. Inhaled long-acting β_2_-agonists added to ICS further improve asthma control and are commonly given as combination inhalers, which improve compliance and control asthma at lower doses of corticosteroids. By contrast, ICS provide much less clinical benefit in COPD and the inflammation is resistant to the action of corticosteroids. This appears to be due to a reduction in HDAC2 activity and expression as a result of oxidative stress. ICS are added to bronchodilators in patients with severe COPD to reduce exacerbations. ICS, which are absorbed from the lungs into the systemic circulation, have negligible systemic side effects at the doses most patients require, although the high doses used in COPD has some systemic side effects and increases the risk of developing pneumonia.

## 1. Introduction

Inhaled corticosteroids (ICS, also known as glucocorticosteroids, glucocorticoids, steroids) are by far the most effective controllers used in the treatment of asthma and the only drugs that can effectively suppress the characteristic inflammation in asthmatic airways, even in very low doses. By contrast, ICS are largely ineffective in suppressing pulmonary inflammation in COPD and have a poor clinical effect. In both asthma and COPD ICS are commonly given as combination inhalers with long-acting β_2_-agonists (LABA).

## 2. Mechanisms of Action

There have been major advances in understanding the molecular mechanisms whereby ICS suppress inflammation in asthma, based on recent developments in understanding the fundamental mechanisms of gene transcription [1,2]. Corticosteroids activate and suppress many genes relevant to understanding their action in asthma (Table 1). Progress has also been made in understanding the molecular mechanisms of corticosteroid resistance in severe asthma and COPD [3].

**Table 1:** Effect of corticosteroids on gene transcription.

***Increased transcription***
• Lipocortin-1
• β_2_-Adrenergic receptors
• Secretory leukocyte inhibitory protein
• IκB-α (inhibitor of NF-κB)
• Anti-inflammatory or inhibitory cytokines
*IL-10, IL-12, IL-1 receptor antagonist*
• Mitogen-activated protein kinase phosphatase-1 (MKP-1, inhibits MAP kinase pathways)

***Decreased transcription***
• Inflammatory cytokines
*IL-2, IL-3, IL-4, IL-5, IL-6, IL-11, IL-13, IL-15, TNFα, GM-CSF, SCF*

• Chemokines
*IL-8, RANTES, MIP-1α, eotaxin*

• Inflammatory enzymes
*Inducible nitric oxide synthase (iNOS), inducible cyclo-oxygenase (COX-2)*
*inducible phospholipase A_2_ (cPLA_2_)*

• Inflammatory peptides
*Endothelin-1*

• Mediator Receptors
*Neurokinin (NK_1_)-, bradykinin (B_2_)-receptors*

• Adhesion molecules
*ICAM-1,VCAM-1*

### 2.1. Cellular Effects

**Figure 1:**
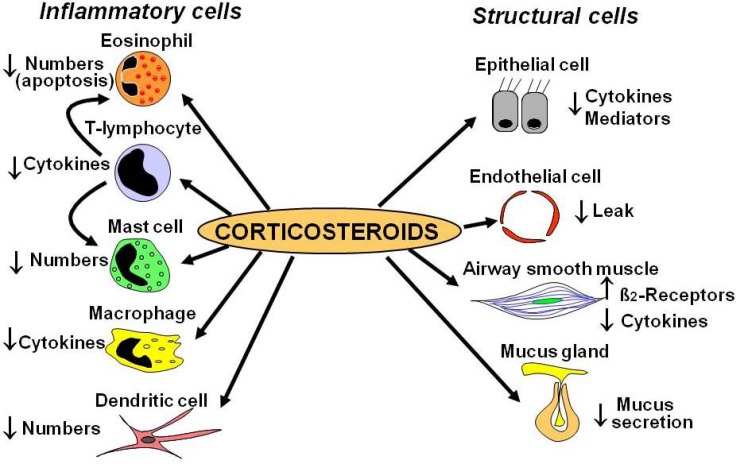
Cellular effect of corticosteroids.

At a cellular level inhaled corticosteroids reduce the numbers of inflammatory cells in asthmatic airways, including eosinophils, T-lymphocytes, mast cells and dendritic cells (Figure 1). 

**Figure 2:**
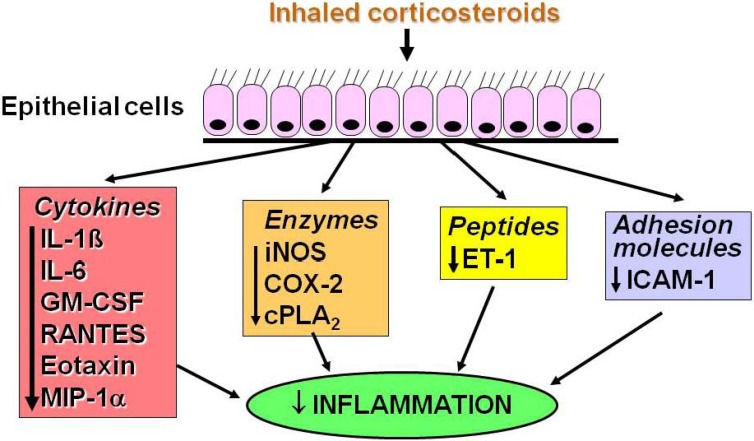
Inhaled corticosteroids may inhibit the transcription of several inflammatory genes in airway epithelial cells and thus reduce inflammation in the airway wall. NF-κB = nuclear factor κB; AP-1 = activator protein-1; GM-CSF = granulocyte-macrophage colony stimulating factor; IL-1 = interleukin-1; iNOS = inducible nitric oxide synthase; NO = nitric oxide; COX-2 = inducible cyclooxygenase; cPLA_2 _= cytoplasmic phospholipase A_2_; PG = prostaglandin; ET = endothelin; ICAM = intercellular adhesion molecule.

These effects of corticosteroids are produced through inhibiting the recruitment of inflammatory cells into the airway by suppressing the production of chemotactic mediators and adhesion molecules and by inhibiting the survival in the airways of inflammatory cells, such as eosinophils, T-lymphocytes and mast cells. Epithelial cells may be a major cellular target for ICS, which are the mainstay of modern asthma management. ICS suppress many activated inflammatory genes in airway epithelial cells (Figure 2). Epithelial integrity is restored by regular ICS. The suppression of mucosal inflammation is relatively rapid with a significant reduction in eosinophils detectable within six hours and associated with reduced airway hyperresponsiveness [4,5,6]. Reversal of airway hyperresponsiveness may take several months to reach a plateau, probably reflecting recovery of structural changes in the airway [7]. 

### 2.2. Glucocorticoid Receptors

**Figure 3:**
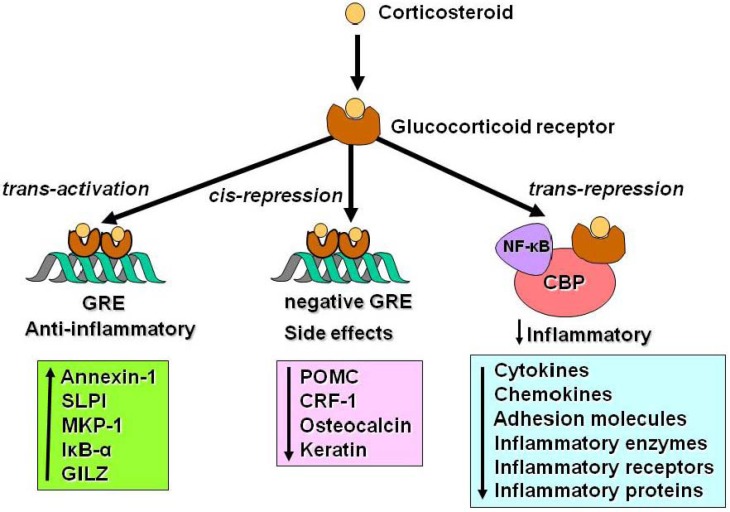
Corticosteroids may regulate gene expression in several ways. Glucocorticoids enter the cell to bind to glucocorticoid receptors in the cytoplasm that translocate to the nucleus. GR homodimers bind to glucocorticoid-response elements (GRE) in the promoter region of steroid-sensitive genes, which may encode anti-inflammatory proteins. Less commonly, GR homodimers interact with negative GREs to suppress genes, particularly those linked to side effects of corticosteroids. Nuclear GR also interact with coactivator molecules, such as CREB-binding protein (CBP), which is activated by proinflammatory transcription factors, such as nuclear factor-κB (NF-κB), thus switching off the inflammatory genes that are activated by these transcription factors. *Other abbreviations:* SLPI: secretory leukoprotease inhibitor; MKP-1: mitogen-activated kinase phosphatase-1; IκB-α: inhibitor of NF-κB; GILZ: glucocorticoid-induced leucine zipper protein; POMC: proopiomelanocortin; CRH: corticotrophin releasing factor.

Corticosteroids diffuse across the cell membrane and bind to glucocorticoid receptors (GR) in the cytoplasm (2). There is only one form of GR that binds corticosteroids termed GRα. GRβ is an alternatively spliced form of GR that interacts with DNA but not with corticosteroids, so may act as a dominant negative inhibitor of glucocorticoid action by interfering with the binding of GR to DNA [8]. Whether GRβ is involved in steroid resistance in asthma is controversial [9]. Activated GRs rapidly translocate to the nucleus where they produce their molecular effects. A pair of GRs (GR homodimer) bind to glucocorticoid response elements (GRE) in the promoter region of steroid-responsive genes and this interaction switches on (and sometimes switches off) gene transcription (Figure 3). Examples of genes that are activated by corticosteroids include genes encoding β_2_-adrenergic receptors and the anti-inflammatory proteins secretory leukoprotease inhibitor and mitogen-activated protein kinase phosphatase-1 (MKP-1) which inhibits MAP kinase pathways. These effects may contribute to the anti-inflammatory actions of corticosteroids [10,11]. GR interaction with negative GREs may suppress gene transcription and it is though that this may be important in mediating many the side effects of corticosteroids. For example, corticosteroids inhibit the expression of osteocalcin that is involved in bone synthesis [12]. 

### 2.3. Switching off Inflammation

**Figure 4:**
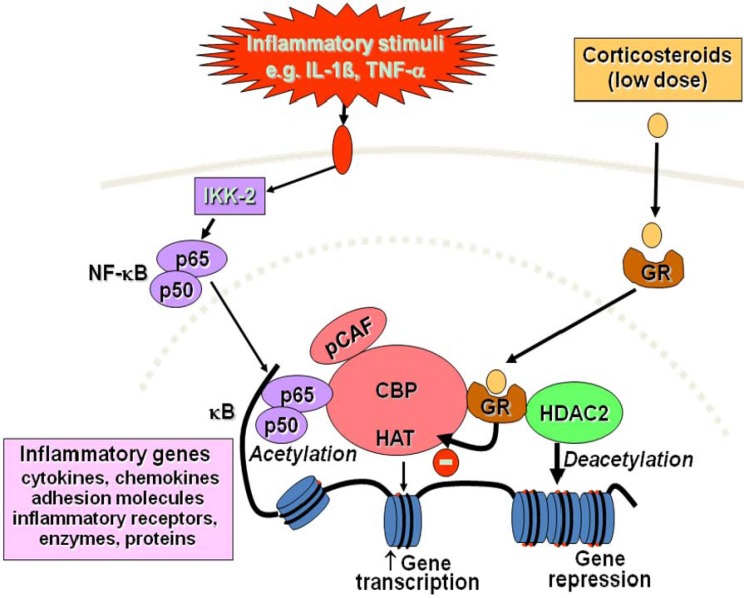
Corticosteroid suppression of activated inflammatory genes. Inflammatory genes are activated by inflammatory stimuli, such as interleukin-1β (IL-1β) or tumour necrosis factor-α (TNF-α), resulting in activation of IKK2 (inhibitor of I-κB kinase-2), which activates the transcription factor nuclear factor κB (NF-κB). A dimer of p50 and p65 NF-κB proteins translocates to the nucleus and binds to specific κB recognition sites and also to coactivators, such as CREB-binding protein (CBP) or p300/CBP-activating factor (pCAF), which have intrinsic histone acetyltransferase (HAT) activity. This results in acetylation of core histone H4, resulting in increased expression of genes encoding multiple inflammatory proteins. Glucocorticoid receptors (GR) after activation by glucocorticoids translocate to the nucleus and bind to coactivators to inhibit HAT activity directly and recruiting histone deacetylase-2 (HDAC2), which reverses histone acetylation leading in suppression of these activated inflammatory genes.

The major action of corticosteroids is to switch off multiple activated inflammatory genes that encode for cytokines, chemokines, adhesion molecules inflammatory enzymes and receptors [1]. These genes are switched on in the airways by proinflammatory transcription factors, such as nuclear factor-κB (NF-κB) and activator protein-1, both of which are activated in asthmatic airways and switch on inflammatory genes by interacting with coactivator molecules, such as CREB-binding protein, that have intrinsic histone acetyltransferase activity. This results in acetylation of core histones, which opens up the chromatin structure so that gene transcription is facilitated [13]. In artificial over-expression systems activated GR may directly interact with NF-κB and AP-1 to inhibit their activity, but this does not appear to occur in asthmatic patients treated with inhaled corticosteroids [14]. Corticosteroid-activated GR also interact with coactivator molecules and this inhibits the interaction of NF-κB with coactivators, thus reducing histone acetylation [1,15]. Reduction of histone acetylation also occurs through the recruitment of histone deacetylase-2 (HDAC2) to the activated inflammatory gene complex by activated GR, thereby resulting in effective suppression of all activated inflammatory genes within the nucleus (Figure 4). This accounts for why corticosteroids are so effective in the control of asthmatic inflammation, but also why they are safe, since other activated genes are not affected.

There may be additional mechanisms that are also important in the anti-inflammatory actions of corticosteroids. Corticosteroids have potent inhibitory effects on mitogen-activated kinase signalling pathways through the induction of MKP-1 and this may inhibit the expression of multiple inflammatory genes [10,11]. Some inflammatory genes, for example granulocyte-macrophage colony stimulating factor, have an unstable messenger RNA that is rapidly degraded by certain RNAses but stabilised when cells are stimulated by inflammatory mediators. Corticosteroids reverse this effect, resulting in rapid degradation of mRNA and reduced inflammatory protein secretion [16]. This may be through the inhibition of proteins that stabilize mRNAs of inflammatory proteins, such as tristretraprolin [17]. 

### 2.4. Corticosteroid Resistance

**Figure 5:**
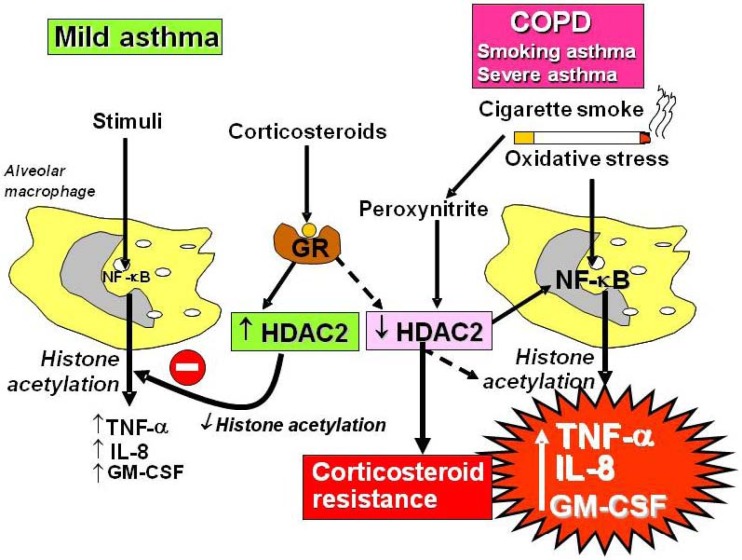
Mechanism of corticosteroid resistance in COPD, smoking asthma and severe asthma. Stimulation of mild asthmatic alveolar macrophages activates nuclear factor-κB (NF-κB) and other transcription factors to switch on histone acetyltransferase leading to histone acetylation and subsequently to transcription of genes encoding inflammatory proteins, such as tumour necrosis factor-α (TNF-α), interleukin-8 (IL-8) and granulocyte-macrophage colony stimulating factor (GM-CSF). Corticosteroids reverse this by binding to glucocorticoid receptors (GR) and recruiting histone deacetylase-2 (HDAC2). This reverses the histone acetylation induced by NF-κB and switches off the activated inflammatory genes. In COPD patients and smoking asthmatics cigarette smoke generates oxidative stress (acting through the formation of peroxynitrite) and in severe asthma and COPD intense inflammation generates oxidative stress to impair the activity of HDAC2. This amplifies the inflammatory response to NF-κB activation, but also reduces the anti-inflammatory effect of corticosteroids, as HDAC2 is now unable to reverse histone acetylation.

Patients with severe asthma have a poor response to corticosteroids, which necessitates the need for high doses and a few patients are completely resistant. All patients with COPD show corticosteroid resistance. Asthmatics who smoke are also relatively corticosteroid-resistant and require increased doses of corticosteroids for asthma control [18]. Several molecular mechanisms have now been identified to account for corticosteroid resistance in severe asthma and COPD [3,19]. In patients with COPD, smoking asthmatics and severe asthma there is a reduction in HDAC2 activity and expression, which prevents corticosteroids switching off activated inflammatory genes (Figure 5)[20,21,22]. In steroid-resistant asthma other mechanisms may also contribute to corticosteroid insensitivity, including reduced translocation of GR as a result of phosphorylation by p38 MAP kinase [23] and abnormal histone acetylation patterns [24]. A proposed mechanism is an increase in GR-β, which prevents GR binding to DNA, but there is little evidence that this would be sufficient to account for corticosteroid insensitivity as the amounts of GR-β are too low [9].

### 2.5. Interaction with β_2_-Adrenergic Receptors

**Figure 6:**
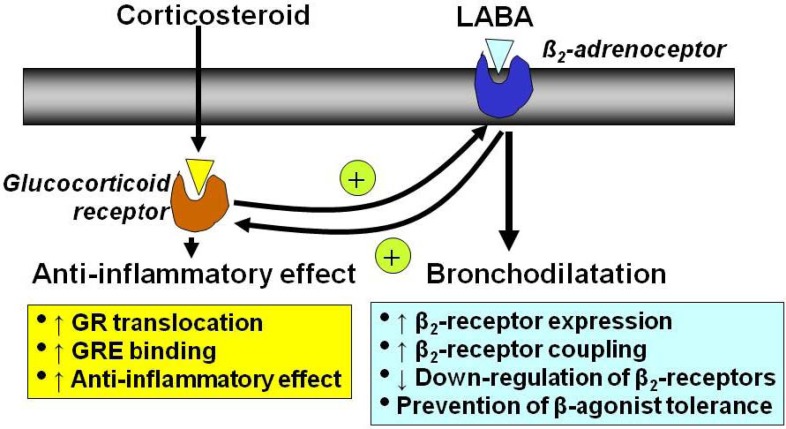
Interaction between corticosteroids and long-acting β_2_-agonists (LABA). Corticosteroids have anti-inflammatory effects but also increase the numbers of β_2_-receptors, whereas β_2_-agonists, as well as inducing direct bronchodilatation, act on glucocorticoid receptors to increase the anti-inflammatory effects of corticosteroids.

Inhaled β_2_-agonists and corticosteroids are frequently used together in the control of asthma and it is now recognized that there are important molecular interactions between these two classes of drug (Figure 6) [25]. Corticosteroids increase the gene transcription of β_2_-receptors, resulting in increased expression of cell surface receptors. This has been demonstrated in human lung *in vitro* [26] and nasal mucosa *in vivo* after topical application of a glucocorticoid [27]. In this way corticosteroids protect against the down-regulation of β_2_-receptors after long-term administration [28]. This may be important for the non-bronchodilator effects of β_2_-agonists, such as mast cell stabilization. Corticosteroids may also enhance the coupling of β_2_-receptors to G-proteins, this enhancing β_2_-agonist effects and reversing the uncoupling of β_2_-receptors that may occur in response to inflammatory mediators, such as interleukin-1β through a stimulatory effect on a G-protein coupled receptor kinase [29].

There is also evidence that β_2_-agonists may affect GR and thus enhance the anti-inflammatory effects of corticosteroids. β_2_-Agonists increase the translocation of GR from cytoplasm to the nucleus after activation by corticosteroids [30]. This effect has now been demonstrated in sputum macrophages of asthmatic patients after an inhaled glucocorticoid and inhaled long-acting β_2_-agonist [31]. This suggests that β_2_-agonists and glucocorticoid enhance each others beneficial effects in asthma therapy. 

## 3. Pharmacokinetics

**Figure 7:**
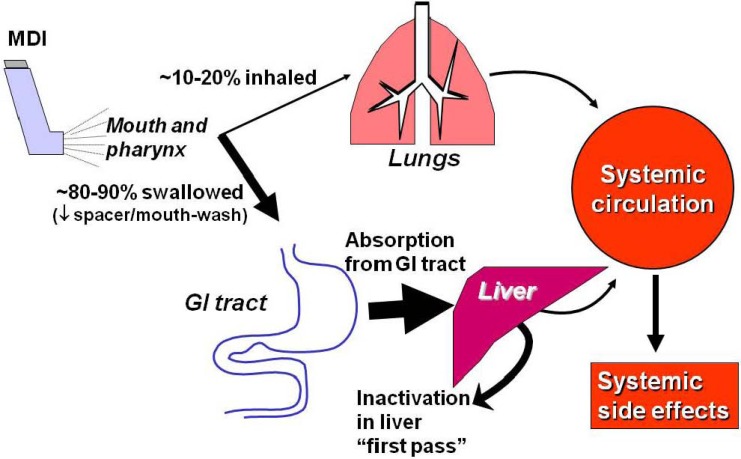
Pharmacokinetics of inhaled glucocorticoids. GI = gastrointestinal

The pharmacokinetics of ICS is important in relation to systemic effects [32,33,34]. The fraction of steroid which is inhaled into the lungs acts locally on the airway mucosa, but may be absorbed from the airway and alveolar surface. This fraction therefore reaches the systemic circulation (Figure 7). The fraction of ICS which is deposited in the oropharynx is swallowed and absorbed from the gut. The absorbed fraction may be metabolized in the liver before reaching the systemic circulation (first-pass metabolism). Budesonide and fluticasone propionate have a greater first pass metabolism than beclomethasone dipropionate (BDP) and are therefore less likely to produce systemic effects at high inhaled doses. The use of a large volume spacer chamber reduces oropharyngeal deposition and therefore reduces systemic absorption of corticosteroids, although this effect is minimal in corticosteroids with a high first pass metabolism [35]. Mouth rinsing and discarding the rinse has a similar effect and this procedure should be used with high dose dry powder steroid inhalers, since spacer chambers cannot be used with these devices. The ideal ICS with optimal therapeutic index should have high lung bioavailability, negligible oral bioavailability, low systemic absorption, high systemic clearance and high protein binding [36].

Ciclesonide is an inactive prodrug that is activated by esterases in the lung to the active metabolite des-ciclesonide [37]. This may reduce oropharyngeal side effects as esterases appear to be less active in this site than in the lower airways. Ciclesonide is also claimed to be effective as a once daily therapy.

## 4. Clinical Use

**Figure 8:**
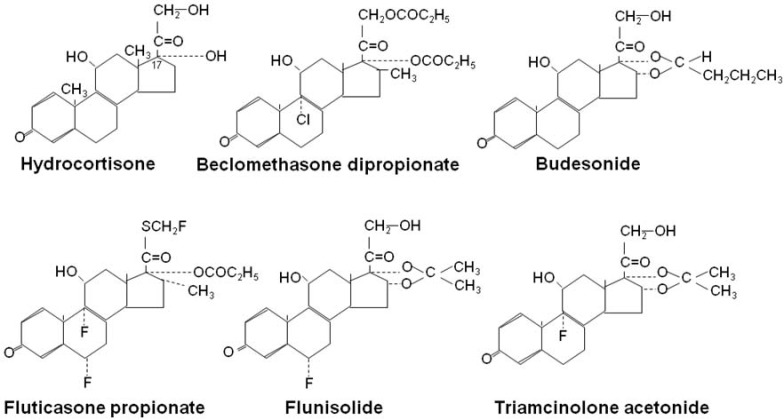
Chemical structures of inhaled glucocorticoids.

There is no doubt that the early use of ICS has revolutionized the management of asthma, with marked reductions in asthma morbidity and improvement in health status. ICS are now recommended as first-line therapy for all patients with persistent asthma [38]. Several topically acting corticosteroids are now available for inhalation (Figure 8). ICS are very effective in controlling asthma symptoms in asthmatic patients of all ages and severity. ICS improve the quality of life of patients with asthma and allow many patients to lead normal lives, improve lung function, reduce the frequency of exacerbations and may prevent irreversible airway changes. They were first introduced to reduce the requirement for oral corticosteroids in patients with severe asthma and many studies have confirmed that the majority of patients can be weaned off oral corticosteroids [3]. By contract ICS are poorly effective in COPD [39].

### 4.1. Use in Asthma

As experience has been gained with ICS they have been introduced in patients with milder asthma, with the recognition that inflammation is present even in patients with mild asthma. Inhaled anti-inflammatory drugs have now become first-line therapy in any patient who needs to use a β_2_-agonist inhaler more than two to three times a week and this is reflected in international guidelines for the management of chronic asthma [38]. In patients with newly-diagnosed asthma an ICS (budesonide 600 µg twice daily) reduced symptoms and β_2_-agonist inhaler usage and improved lung function. These effects persisted over the two years of the study, whereas in a parallel group treated with inhaled β_2_-agonists alone there was no significant change in symptoms or lung function [40]. In another study patients with mild asthma treated with a low dose of ICS (budesonide 400 µg daily) showed less symptoms and a progressive improvement in lung function over several months and many patients became completely asymptomatic [41]. There was also a significant reduction in the number of exacerbations; in patients with mild asthma a low dose of corticosteroids (budesonide 400 µg daily) significantly reduces exacerbation by around 40% over a three year period [42]. Although the effects of ICSs on AHR may take several months to reach a plateau, the reduction in asthma symptoms occurs much more rapidly and reduced inflammation is seen within hours [4,5,6]. High dose ICS may be used for the control of more severe asthma. This markedly reduces the need for maintenance oral corticosteroids [43]. With the use of add-on therapies, particularly long-acting β_2_-agonists (LABA), most patients can now be controlled on much lower doses of ICS so that high doses are needed in only a few patients with severe disease. ICS are the treatment of choice in nocturnal asthma, which is a manifestation of inflamed airways, reducing nocturnal awakening and reducing the diurnal variation in airway function. ICS effectively control asthmatic inflammation but must be taken regularly. When ICS are discontinued there is usually a gradual increase in symptoms and airway responsiveness back to pretreatment values. Reduction in the dose of ICS is associated with an increase in symptoms and this is preceded by an increase in exhaled NO and sputum eosinophils [44,45]. ICS are equally effective in children. Nebulized budesonide reduces the need for oral corticosteroids and also improved lung function in children under the age of three [46]. ICS given *via* a large volume spacer improve asthma symptoms and reduce the number of exacerbations in preschool children and in infants.

### 4.2. Dose-Response Studies

Surprisingly, the dose-response curve for the clinical efficacy of ICS is relatively flat and, while all studies have demonstrated a clinical benefit of ICS, it has been difficult to demonstrate differences between doses, with most benefit obtained at the lowest doses used [47,48]. This is in contrast to the steeper dose-response for systemic effects, implying that while there is little clinical benefit from increasing doses of ICS the risk of adverse effects is increased. However, the dose-response effect of ICS may depend on the parameters measured and, while it is difficult to discern a dose-response when traditional lung function parameters are measured, there may be a dose-response effect in prevention of asthma exacerbations. Thus, there is a significantly greater effect of budesonide 800 μg daily compared to 200 μg daily in preventing severe and mild asthma exacerbations [49]. Normally, a four-fold or greater difference in dose has been ­required to detect a statistically significant (but often small) difference in effect on commonly measured outcomes such as symptoms, PEF, use of rescue β_2_-agonist and lung function and even such large differences in dose are not always associated with significant differences in response. These findings suggest that pulmonary function tests or symptoms may have a rather low sensitivity in the assessment of the effects of ICS. This is obviously important for the interpretation of clinical comparisons between different ICS or inhalers. It is also important to consider the type of patient included in clinical studies. Patients with relatively mild asthma may have relatively little room for improvement with ICS, so that maximal improvement is obtained with relatively low doses. Patients with more severe asthma or with unstable asthma may have more room for improvement and may therefore show a greater response to increasing doses, but it is often difficult to include such patients in controlled clinical trials.

More studies are needed to assess whether other outcome measures such as AHR or more direct measurements of inflammation, such as sputum eosinophils or exhaled NO, may be more sensitive than traditional outcome measures such as symptoms or lung function tests [50,51,52,53]. Higher doses of ICS are needed to control AHR than to improve symptoms and lung function, and this may have a better long-term outcome in terms of reduction in structural changes of the airways [54]. Measurement of sputum eosinophils to adjust the dose of ICS may reduce the overall dose requirement for ICS and exacerbations [55,56]. Monitoring of exhaled NO may also reduce the requirement for corticosteroids but is not yet practical in clinical practice [57].

### 4.3. Prevention of Irreversible Airway Changes in Asthma

Some patients with asthma develop an element of irreversible airflow obstruction, but the pathophysiological basis of these changes is not yet understood. It is likely that they are the result of chronic airway inflammation and that they may be prevented by treatment with ICS. There is some evidence that the annual decline in lung function may be slowed by the introduction of ICS [58] and this is supported by a five year study of low dose budesonide in patients with mild asthma [59,60]. Increasing evidence also suggests that delay in starting ICS may result in less overall improvement in lung function in both adults and children [61,62,63]. These studies suggest that introduction of ICS at the time of diagnosis is likely to have the greatest impact [62,63]. So far there is no evidence that early use of ICS is curative and even when ICS are introduced at the time of diagnosis, symptoms and lung function revert to pretreatment levels when corticosteroids are withdrawn [61]. 

### 4.4. Reduction in Mortality

In a retrospective review of the risk of mortality and prescribed anti-asthma medication, there was a significant protection provided by regular ICS therapy [64]. By contrast, asthma mortality appears to increase with increasing usage of short-acting β_2_-agonists, reflecting the fact that increased rescue therapy is a marker of poor asthma control [65]. The increase in use of rescue therapy should result in an increase in the maintenance dose of ICS. The long-acting inhaled β_2_-agonist salmeterol is associated with a small increase in asthma mortality, but the excess deaths appear to be related to underuse of ICS [66,67].

### 4.5. Comparison between ICS

Several ICS are currently on the market for use in asthma, although their availability varies between countries. There have been relatively few studies comparing efficacy of the different ICS, and it is important to take into account the delivery system and the type of patient under investigation when such comparisons are made. Because of the relatively flat dose-response curve for the clinical parameters normally used in comparing doses of ICS, it may be difficult to see differences in efficacy of ICS. Most comparisons have concentrated on differences in systemic effects at equally efficacious doses, although it has often proved difficult to establish dose-equivalence [68]. There are few studies comparing different doses of ICS in asthmatic patients. Budesonide has been compared with BDP and in adults and children it appears to have comparable anti-asthma effects at equal doses, whereas fluticasone propionate (FP) appears to be approximately twice as potent as BDP and budesonide [48]. Studies have consistently shown that FP and budesonide have less systemic effects than BDP, triamcinolone and flunisolide [33]. The newer ICS mometasone and ciclesonide are claimed to have less systemic effects [69,70]. 

### 4.6. Clinical Application in Asthma Patients

ICS are now recommended as first-line therapy for all patients with persistent symptoms. ICS should be started in any patient who needs to use a ß_2_-agonist inhaler for symptom control more than three times weekly. It is conventional to start with a low dose of ICS and to increase the dose until asthma control is achieved. However, this may take time and a preferable approach is to start with a dose of corticosteroids in the middle of the dose range (400 µg budesonide or equivalent twice daily) to establish asthma control. Once control is achieved (defined as normal or best possible lung function and infrequent need to use an inhaled β_2_-agonist) the dose of inhaled corticosteroid should be reduced in a step-wise manner to the lowest dose needed for optimal control. It may take as long as three months to reach a plateau in response and any changes in dose should be made at intervals of three months or more. When daily doses of ≥800 µg daily are needed a large volume spacer device should be used with a metered dose inhaler (MDI) and mouth washing with a dry powder inhaler in order to reduce local and systemic side effects. ICS are usually given as a twice daily dose in order to increase compliance. When asthma is unstable four times daily dosage may be preferable [71].

The dose of inhaled corticosteroid should be increased to 2,000 µg daily if necessary, but higher doses may result in systemic effects. It may be preferable to add a low dose of oral corticosteroid, since higher doses of ICS are expensive and have a high incidence of local side effects. Nebulized budesonide has been advocated in order to give an increased dose of inhaled corticosteroid and to reduce the requirement for oral corticosteroids [72], but this treatment is expensive and may achieve its effects largely *via* systemic absorption. The dose of inhaled corticosteroid should be the minimal dose that controls asthma and once control is achieved the dose should be slowly reduced [73].

### 4.7. Use in COPD

Patients with COPD have a poor response to corticosteroids in comparison to asthma with little improvement in lung function [39]. High doses of ICS have consistently been shown a reduction (20–25%) in exacerbations in patients with severe disease and this is the main clinical indication for their use [74]. However, several large studies have shown that corticosteroids fail to reduce the progression in COPD (measured by annual fall in FEV_1_) [75] and they have not been found to reduce mortality in a large study [76]. These results are likely to reflect the resistance of pulmonary inflammation to corticosteroids in COPD patients as a result of the reduction in HDAC2 [22]. 

## 5. Add-on Therapy

Previously it was recommended to increase the dose of ICS if asthma was not controlled, on the assumption that there was residual inflammation of the airways. However the dose response effect of ICS is relatively flat, so that there is little improvement in lung function after increasing the dose of ICS. An alternative strategy is to add some other class of controller drug and this is more effective than increasing the dose of ICS for most patients [77]. 

### 5.1. Long-Acting β_2_-Agonists

In patients who are not controlled on BDP 200 μg twice daily, addition of salmeterol 50 μg twice daily was more effective than increasing the dose of inhaled corticosteroid to 500 μg twice daily, in terms of lung function improvement, use of rescue β_2_-agonist use and symptom control [78]. This has been confirmed in several other studies [79]. Similar results have been found with another long-acting inhaled β_2_-agonist formoterol, which in addition reduced the frequency of mild and severe asthma exacerbations in patients with mild, moderate and severe persistent asthma [49,80]. Analysis of several studies clearly show that adding a LABA is more effective than increasing the dose of ICS in terms of improving asthma control and reducing exacerbations [81]. These studies showing the great efficacy of combined corticosteroids and LABA compared to increased doses of LABA have led to the development of fixed combinations of corticosteroids and long-acting β_2_-agonists, such as FP/salmeterol and budesonide/formoterol, which may be more convenient for patients. These fixed combination inhalers also ensure that patients do not discontinue their ICS when a long-acting bronchodilator is used. For patients with mild persistent asthma combination inhalers are no more effective than the ICS alone in controlled trials [82], but may have an advantage in the real world where adherence to regular ICS is very low.

Recently studies have demonstrated that when formoterol combined with budesonide is used as a reliever therapy this gives better control of asthma compared to the normally used short-acting β_2_-agonist as a rescue therapy with either the same dose of combination inhaler or a high dose of ICS as maintenance treatment [83,84]. This advantage is particularly striking in terms of reducing the number of severe exacerbations. When formoterol was used as the reliever therapy this reduce exacerbations to a greater extent than the short-acting β_2_-agonist terbutaline but the combination was even more effective [85]. This suggests that the “as required” use of ICS contributes to the marked reduction in acute exacerbations. The mechanisms by which corticosteroids as required improve asthma control and reduce exacerbations are not completely understood, but exacerbations of asthma evolve over several days when patients take increasing amounts of rescue medication [86]. During this time there is increasing inflammation of the airways, as may be measure by exhaled nitric oxide and sputum eosinophils [44]. Taking the inhaled corticosteroid at the same time as the formoterol to relieve symptoms may suppress this evolving inflammation, particularly since corticosteroids appear to have a relatively rapid onset of effect in suppressing airway inflammation [87]. 

LABA/corticosteroid inhalers are also more effective in COPD patients than either treatment alone and reduce exacerbations and improve symptoms [88,89]. They are more effective than LABA alone in reducing exacerbations [90]. This may be explained by molecular interactions between LABA and corticosteroids as discussed above. However, there is a reduction in COPD mortality although this fails to reach significance in one large study (TORCH) [76], although this has been seen in another smaller study (INSPIRE) [91].

### 5.2. Theophylline

Addition of low doses of theophylline (giving plasma concentrations of <10 mg/L) are more effective than doubling the dose of inhaled budesonide, either in mild or severe asthma [92,93,94]. However this is less effective than using a long-acting inhaled β_2_-agonist as add-on therapy [95]. Theophylline has not been examined as an add-on therapy in COPD patients but there are theoretical reasons to believe that low dose theophylline may reverse corticosteroid resistance in COPD.

### 5.3. Anti-Leukotrienes

Anti-leukotrienes have also been used as an add-on therapy in asthma [96,97], although this is less effective than addition of long-acting β_2_-agonists [98,99,100]. There is no role for anti-leukotrienes in COPD patients.

## 6. Side Effects

The efficacy of ICS is now established in short- and long-term studies in adults and children, but there are still concerns about side effects, particularly in children and when high inhaled doses are used. Several side effects have been recognized (Table 2).

**Table 2:** Side effects of inhaled corticosteroids.

**Local side effects**
Dysphonia
Oropharyngeal candidiasis
Cough
Pneumonia (COPD patients)
**Systemic side effects**
Adrenal suppression
Growth suppression
Bruising
Osteoporosis
Cataracts
Glaucoma
Metabolic abnormalities (glucose, insulin, triglycerides)
Psychiatric disturbances

### 6.1. Local Side Effects

Side effects due to the local deposition of the ICS in the oropharynx may occur with steroid, but the frequency of complaints depends on the dose and frequency of administration and on the delivery device used. The commonest complaint is of hoarseness of the voice (dysphonia) and may occur in over 50% of patients using MDI. Dysphonia is not appreciably reduced by using spacers, but may be less with dry powder devices. Dysphonia may be due to myopathy of laryngeal muscles and is reversible when treatment is withdrawn [101]. For most patients it is not troublesome but may be disabling in singers and lecturers. Oropharyngeal candidiasis (thrush) may be a problem in some patients, particularly in the elderly, with concomitant oral corticosteroids and more than twice daily administration [102]. Large volume spacer devices protect against this local side effect by reducing the dose of ICS that deposits in the oropharynx.

### 6.2. Infections

There is no evidence that ICS, even in high doses, increase the frequency of infections, including tuberculosis, in the lower respiratory tract in asthmatic patients. Recently several large controlled studies have shown that high dose ICS increase physician-diagnosed pneumonias, either used alone or in combination with a LABA [75,90] and this has been confirmed in an epidemiological study of hospital admissions for pneumonia amongst COPD patients [103]. The mechanism of pneumonia in COPD is uncertain, but is apparently seen more often with FP than with budesonide [104].

### 6.3. Systemic Side Effects

The efficacy of ICS in the control of asthma is undisputed, but there are concerns about systemic effects of ICS, particularly as they are likely to be used over long periods and in children of all ages [33,105]. The safety of ICS has been extensively investigated since their introduction 30 years ago [32]. One of the major problems is to decide whether a measurable systemic effect has any significant clinical consequence and this necessitates careful long-term follow-up studies. As biochemical markers of systemic corticosteroid effects become more sensitive, then systemic effects may be seen more often, but this does not mean that these effects are clinically relevant. There are several case reports of adverse systemic effects of ICS, and these may be idiosyncratic reactions, which may be due to abnormal pharmacokinetic handing of the inhaled corticosteroid. The systemic effect of an inhaled corticosteroid will depend on several factors, including the dose delivered to the patient, the site of delivery (gastrointestinal tract and lung), the delivery system used and individual differences in the patient's response to the corticosteroid. Recent studies suggest that systemic effects of inhaled corticosteroid are less in patients with more severe asthma, presumably as less drug reaches the lung periphery [106,107]. The systemic effect of an ICS is dependent on the amount of drug absorbed into the systemic circulation. Approximately 80–90% of the inhaled dose from an MDI deposits in the oropharynx and is swallowed and subsequently absorbed from the gastrointestinal tract. Use of a large volume spacer device markedly reduces the oropharyngeal deposition, and therefore the systemic effects of ICS, although thus is less important when oral bioavailability is minimal, as with FP. For dry powder inhalers similar reductions in systemic effects may be achieved with mouth-washing and discarding the fluid. All patients using a daily dose of ≥800 µg of an inhaled corticosteroid should therefore use either a spacer or mouth washing to reduce systemic absorption. Approximately 10% of an MDI enters the lung and this fraction (which presumably exerts the therapeutic effect) may be absorbed into the systemic circulation. As the fraction of ICS deposited in the oropharynx is reduced, the proportion of the inhaled dose entering the lungs is increased. More efficient delivery to the lungs is therefore accompanied by increased systemic absorption, but this is offset by a reduction in the dose needed for optimal control of airway inflammation. For example, a multiple dry powder delivery system, the Turbuhaler, delivers approximately twice as much corticosteroid to the lungs as other devices, and therefore has increased systemic effects. However this is compensated for by the fact that only half the dose is required.

*Adrenal Suppression*. Corticosteroids may cause hypothalamic-pituitary-adrenal (HPA) axis suppression by reducing corticotrophin (ACTH) production, which reduces cortisol secretion by the adrenal gland. The degree of HPA suppression is dependent on dose, duration, frequency and timing of corticosteroid administration. Measurement of HPA axis function provides evidence for systemic effects of an inhaled corticosteroid. Basal adrenal cortisol secretion may be measured by a morning plasma cortisol, 24h urinary cortisol or by plasma cortisol profile over 24 h. Other tests measure the HPA response following stimulation with tetracosactrin (which measures adrenal reserve) or stimulation with metyrapone and insulin (which measure the response to stress). There are many studies of HPA axis function in asthmatic patients with ICS, but the results are inconsistent as they have often been uncontrolled and patients have also been taking courses of oral corticosteroids (which may affect the HPA axis for weeks) [32]. BDP, budesonide and FP at high doses by conventional MDI (>1600 µg daily) give a dose-related decrease in morning serum cortisol levels and 24 h urinary cortisol, although values still lie well within the normal range. However, when a large volume spacer is used doses of 2000 µg daily of BDP or budesonide have little effect on 24 h urinary cortisol excretion. Stimulation tests of HPA axis function similarly show no consistent effects of doses of 1500 µg or less of inhaled corticosteroid. At high doses (>1500 µg daily) budesonide and FP have less effect than BDP on HPA axis function. In children no suppression of urinary cortisol is seen with doses of BDP of 800µg or less. In studies where plasma cortisol has been measured at frequent intervals there was a significant reduction in cortisol peaks with doses of inhaled BDP as low as 400 µg daily, although this does not appear to be dose-related in the range 400–1000 µg . The clinical significance of these effects is not certain, however. 

*Bone Metabolism*. Corticosteroids lead to a reduction in bone mass by direct effects on bone formation and resorption and indirectly by suppression of the pituitary-gonadal and HPA axes, effects on intestinal calcium absorption, renal tubular calcium reabsorption and secondary hyperparathyroidism [108]. The effects of oral corticosteroids on osteoporosis and increased risk of vertebral and rib fractures are well known, but there are no reports suggesting that long-term treatment with ICS is associated with an increased risk of fractures. Bone densitometry has been used to assess the effect of ICS on bone mass. Although there is evidence that bone density is less in patients taking high dose ICS, interpretation is confounded by the fact that these patients are also taking intermittent courses of oral corticosteroids. Changes in bone mass occur very slowly and several biochemical indices have been used to assess the short-term effects of ICS on bone metabolism. Bone formation has been measured by plasma concentrations of bone-specific alkaline phosphatase, serum osteocalcin or procollagen peptides. Bone resorption may be assessed by urinary hydroxyproline after a 12 hours fast, urinary calcium excretion and pyridinium cross-link excretion. ICS, even at doses up to 2,000 µg daily, have no significant effect on calcium excretion, but acute and reversible dose-related suppression of serum osteocalcin has been reported with BDP and budesonide when given by conventional MDI in several studies. Budesonide consistently has less effect than BDP at equivalent doses and only BDP increases urinary hydroxyproline at high doses. With a large volume spacer even doses of 2,000 µg daily of either BDP or budesonide are without effect on plasma osteocalcin concentrations, however. Urinary pyridinium and deoxypyridinoline cross-links, which are a more accurate and stable measurement of bone and collagen degradation, are not increased with ICS (BDP > 1,000 µg daily), even with intermittent courses of oral corticosteroids. It is important to monitor changes in markers of bone formation as well as bone degradation, as the net effect on bone turnover is important. There is no evidence that ICS increase the frequency of fractures. Long-term treatment with high dose ICS has not been associated with any consistent change in bone density. Indeed, in elderly patients there may be an increase in bone density due to increased mobility.

*Connective Tissue Effects*. Oral and topical corticosteroids cause thinning of the skin, telangiectasiae and easy bruising, probably as a result of loss of extracellular ground substance within the dermis, due to an inhibitory effect on dermal fibroblasts. There are reports of increased skin bruising and purpura in patients using high doses of inhaled BDP, but the amount of intermittent oral corticosteroids in these patients is not known. Easy bruising in association with ICS is more frequent in elderly patients [109] and there are no reports of this problem in children. Long-term prospective studies with objective measurements of skin thickness are needed with different ICS.

*Cataracts*. Long-term treatment with oral corticosteroids increase the risk of posterior subcapsular cataracts and there are several case reports describing cataracts in individual patients taking ICS [32]. In a recent cross-sectional study in patients aged 5–25 years taking either inhaled BDP or budesonide no cataracts were found on slit-lamp examination, even in patients taking 2,000 µg daily for over 10 years [110]. A slight increase in the risk of glaucoma in patient staking very high does of inhaled corticosteroids has also been identified [111].

*Growth*. There has been particular concern that ICS may cause stunting of growth and several studies have addressed this issue. Asthma itself (as with other chronic diseases) may have an effect on the growth pattern and has been associated with delayed onset of puberty and decceleration of growth velocity that is more pronounced with more severe disease [112]. However, asthmatic children appear to grow for longer, so that their final height is normal. The effect of asthma on growth make it difficult to assess the effects of ICS on growth in cross-sectional studies, particularly as courses of oral corticosteroids is a confounding factor. Longitudinal studies have demonstrated that there is no significant effect of ICS on statural growth in doses of up to 800 µg daily and for up to five years of treatment (32).A meta-analysis of 21 studies, including over 800 children, showed no effect of inhaled BDP on statural height, even with higher doses and long duration of therapy [113] and in a large study of asthmatics treated with ICS during childhood there was no difference in statural height compared to normal children [114]. Another long-term follow-up study showed no effect of corticosteroids on final height in children treated over several years [115]. Short-term growth measurements (knemometry) have demonstrated that even a low dose of an oral corticosteroid (prednisolone 2.5 mg) is sufficient to give complete suppression of lower leg growth. However inhaled budesonide up to 400 µg is without effect, although some suppression is seen with 800 µg and with 400 µg BDP. The relationship between knemometry measurements and final height are uncertain since low doses of oral corticosteroid that have no effect on final height cause profound suppression.

*Metabolic Effects.* Several metabolic effects have been reported after ICS, but there is no evidence that these are clinically relevant at therapeutic doses. In adults fasting glucose and insulin are unchanged after doses of BDP up to 2,000 µg daily and in children with inhaled budesonide up to 800 µg daily. In normal individuals high dose inhaled BDP may slightly increase resistance to insulin. However, in patients with poorly controlled asthma high doses of BDP and budesonide paradoxically decrease insulin resistance and improve glucose tolerance, suggesting that the disease itself may lead to abnormalities in carbohydrate metabolism. Neither BDP 2,000 µg daily in adults nor budesonide 800 µg daily in children have any effect on plasma cholesterol or triglycerides.

*Psychiatric Effects.* There are various reports of psychiatric disturbance, including emotional liability, euphoria, depression, aggressiveness and insomnia, after ICS. Only eight such patients have so far been reported, suggesting that this is very infrequent and a causal link with ICS has usually not been established.

*Pregnancy.* Based on extensive clinical experience ICS appear to be safe in pregnancy, although no controlled studies have been performed. There is no evidence for any adverse effects of ICS on the pregnancy, the delivery or on the foetus [116]. It is important to recognise that poorly controlled asthma may increase the incidence of perinatal mortality and retard intra-uterine growth, so that more effective control of asthma with ICS may reduce these problems.

*Side Effects in COPD*. Patients with COPD are elderly and are likely to have increased systemic side effects from ICS as they have several additional risk factors. There have been fewer studies of systemic side effects in COPD patients. However, a systematic review found no reduction in bone mineral density or increase in fractures in COPD patients treated for up to 3 years with ICS [75]. An epidemiological study showed an increase in cataracts which are more common in an elderly population [117]. Many patients with COPD suffer from co-morbidities, including hypertension, metabolic syndrome and diabetes, and may therefore have a worsening of these conditions, but this has not yet been systematically investigated.
